# Interfacial Polymerization on Polyethersulfone Ultrafiltration Membrane to Prepare Nanofiltration Layers for Dye Separation

**DOI:** 10.3390/polym15092018

**Published:** 2023-04-24

**Authors:** Lulu Liu, Weilin Wu, Xiaogang Jin, Xiong Luo, Lili Wu

**Affiliations:** 1 School of Materials Science and Engineering, Wuhan University of Technology, Wuhan 430070, China; 303786@whut.edu.cn (L.L.); valeeraing@163.com (X.L.); 2 School of Pharmaceutical Sciences, Hunan University of Medicine, No.492 South Jinxi Road, Huaihua 418000, China; wuweilin1005@163.com

**Keywords:** interfacial polymerization, surface modification, dye separation, tannic acid

## Abstract

Nanofiltration membranes are of great significance to the treatment of dye wastewater. Interfacial polymerization is a widely used method to fabricate nanofiltration membranes. In this study, the interaction of tannic acid-assisted polyethylene polyamine (PEPA) with terephthalaldehyde (TPAL) was performed on PES ultrafiltration membranes using novel nitrogen-rich amine monomers and relatively less reactive aldehyde-based monomers. A new nanofiltration membrane ((T-P-T)/PES) was prepared by interfacial polymerization. Fourier transform infrared spectroscopy, X-ray photoelectron spectroscopy, and scanning electron microscopy were used to analyze the elemental composition, bonding state, and surface morphology of the membrane surface. The effects of the PEPA deposition time, TPAL concentration, interfacial reaction time, and curing time on the nanofiltration layer were investigated. The modified membrane, prepared under optimal conditions, showed strong dye separation ability. The permeation of the modified membrane could reach 68.68 L·m^−2^·h^−1^·bar^−1^, and the rejection of various dyes was above 99%. In addition, the (T-P-T)/PES membrane showed good stability during long-term dye separation.

## 1. Introduction

With the continuous development of the global textile industry, water pollution from the textile industry has become more and more serious, which has caused major environmental problems [1,2,3]. The dye wastewater produced in the production process of the printing industry has high chromaticity, complex components, and poor biodegradability, which poses a serious threat to human health and aquatic organisms. It is a kind of harmful industrial wastewater that is difficult to treat [4,5,6]. Therefore, it is of great significance to properly treat textile wastewater and reduce the discharge of such highly polluted wastewater.

To date, several methods for the removal of dye wastewater have been applied for the treatment of dye wastewater. The existing methods include adsorption [7], photocatalytic degradation, chemical degradation [8,9], biodegradation [10], and membrane filtration [11,12,13]. Each method has its own advantages and limitations in terms of cost, efficiency, feasibility, and environmental impact. Compared with traditional dye separation technology, membrane separation technology has the advantages of high energy efficiency, simple operating conditions, low cost, and environmental friendliness and is increasingly being used in water treatment [14,15]. Polyethersulfone (PES) membrane is an excellent membrane separation material for water purification and recovery due to its good mechanical properties and chemical stability [16,17], and it is widely used in dye separation.

Currently, interfacial polymerization (IP) is a widely used method to prepare composite nanofiltration membranes [18,19]. Polyamide selective layers made from the reactive monomers piperazine (PIP) and trimethylbenzene chloride (TMC) by interfacial polymerization have dense structures and relatively low membrane fluxes [20]. Membranes formed by interfacial polymerization are also widely used for dye separation [21,22]. According to the formation mechanism of interfacial polymerization membranes, the reactive monomer has a fundamental and significant effect on the structure and separation properties of polymer layers [12,23]. Therefore, suitable monomer materials must be selected to prepare nanofiltration membranes with improved permeability.

Inspired by the remarkable and pervasive adhesive ability of marine mussels on various substrates, the oxidative polymerization of dopamine (DA) into polydopamine (PDA) coatings has become a common method for surface modification [24,25,26,27]. However, for industrial manufacturing purposes, DA is undesirable because it degrades rapidly in an aerobic environment, thus requiring stringent storage conditions. Moreover, DA has a high cost, and these factors limit its large-scale applications [28]. Tannic acid (TA) is derived from polyphenols [29,30], and it has similar adhesion properties and densification as PDA coatings [31,32,33]. TA contains catechol and a galloyl group and has various physical and chemical properties, such as absorbing ultraviolet rays, scavenging free radicals, chelating metal ions, and antibacterial properties. It can adhere to the surface of various substrates through covalent and/or non-covalent bonding forces [34,35]. Overall, the ubiquitous application of TA in membrane preparation is mainly attributed to its strong surface-binding affinity, coating formation through covalent interactions with nucleophiles [36,37,38], or chelation with metal ions [39,40,41].

Polyethylene polyamine (PEPA) is an amino-rich hydrophilic substance with good surface activity and temperature resistance. Due to its good hydrophilicity, it is mainly used in the preparation of crude oil emulsion breakers, sewage treatment agents, ion exchange membranes, etc. In addition, the amino density of PEPA is similar to that of tetraethylenepentamine (TEPA) and polyethyleneimine (PEI), but it is less expensive [42]. The interfacial polymerization of PEPA with terephthalaldehyde (TPAL) was chosen to generate a nanofiltration layer on the PES membrane due to the relatively low reaction rate of the amino and aldehyde groups and the easier control of the interfacial reaction time.

However, the poor adhesion of the PEPA causes an unstable coating to form on the film surface. Therefore, a “chain” is needed to connect the PEPA to the substrate. TA can easily react with the abundant amino groups on the PEPA molecular chain to form a hydrophilic and stable crosslink. PEPA can react with TA via Michael addition or Schiff base, which facilitates the uniform deposition of the PEPA on the substrate [43].

**Figure 1:**
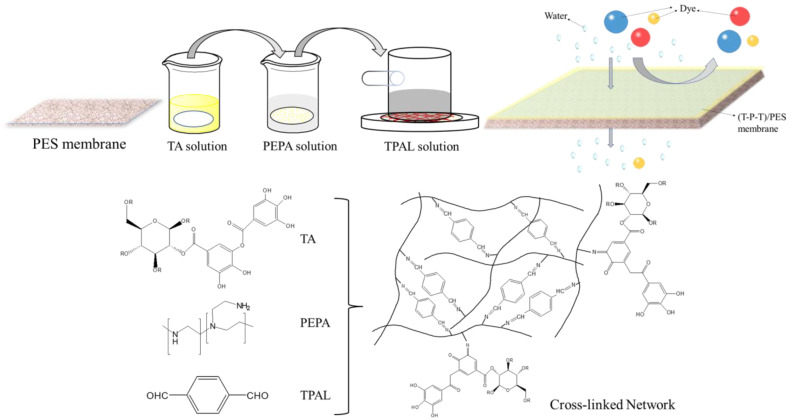
The coating schematic diagram of modified membrane.

In this study, we prepared selective layers with enhanced flux and efficient dye separation on PES ultrafiltration membranes by interfacial polymerization of PEPA and TPAL using TA as an auxiliary modifier. The coating schematic is shown in Figure 1. In this reaction, TA can be used as a binder to increase the aggregation of PEPA on the membrane surface or as a modifier of interfacial polymerization by interacting with PEPA to reduce the distribution and diffusion of PEPA, thus changing the morphology and structure. The properties of the prepared membranes were evaluated, such as the surface morphology, chemical elements, and hydrophilicity. In addition, better-performing nanofiltration membranes could be obtained by adjusting the monomer concentration, interfacial reaction time, and curing time. The separation performance and stability of the modified membranes were systematically evaluated.

## 2. Materials and Methods

Polyethersulfone (PES) ultrafiltration membranes (UF0302) were purchased from Guochu Technology (Xiamen) Co., Ltd. (Xiamen, China). Tannic acid (TA 98%), polyvinylpolyamine (PEPA Mw = 273 g·mol^−1^), terephthalaldehyde (TPAL), n-hexane, and ethanol were purchased from Aladdin. Tris(hydroxymethyl)aminomethane (Tris) and hydrochloric acid (HCl) were provided by Sinopharm Chemical Reagent Co., Ltd. Methyl blue (MB, 99%), Congo red (CR, 99%), and Rose bengal (RB, 95%) were purchased from Ron’s reagent; methyl orange (MO, 96%), Alcian blue 8GX (ACB, ≥50%), and Coomassie brilliant blue G250 (BBG, AR) were purchased from Aladdin (as shown in Table 1). Deionized water was used in the experiments, and all the other reagents were analytical-grade reagents and could be used without further purification.

**Figure 2:**
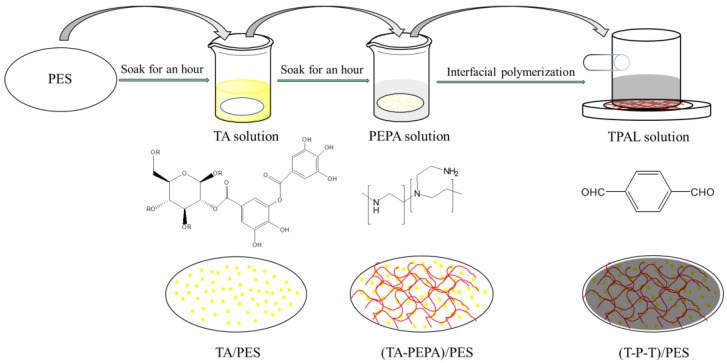
Preparation of (TA-PEPA-TPAL)/PES membrane.

A schematic diagram of the synthesis process of the composite nanofiltration membrane is shown in Figure 2. First, 30 mg of TA was dissolved in 100 mL of Tris-HCl buffer solution (adjusted to pH 7.8 with HCl), and then the PES ultrafiltration membrane was soaked in TA solution for 1 h and removed, which was recorded as TA/PES. The membranes were washed 3–5 times with deionized water to remove the excess TA from the membrane surface. Subsequently, the TA/PES membranes were immersed in an aqueous PEPA solution at a concentration of 0.3 g·L^−1^ for 0.5–12 h, noted as (TA-PEPA)/PES. The membranes were washed 3–5 times with deionized water to remove the excess PEPA from the membrane surface. Subsequently, 10 mg–50 mg of TPAL was dissolved in 50 mL of hexane, and the (TA-PEPA)/PES membrane was immersed in the hexane solution for 10–40 min, noted as (T-P-T)/PES. It is worth noting that only the upper surface of the membrane was immersed in the TPAL solution. After the reaction was completed, the membrane surface was rinsed with hexane 3–5 times to remove the excess TPAL. Finally, the membrane was cured at 40 °C for 2.5 or 10 min.

The chemical composition of the modified membrane was characterized by attenuated total reflection Fourier transform infrared spectroscopy (ATR-FTIR, Thermo Nicolet, Waltham, MA, USA), X-ray photoelectron spectroscopy (XPS, ESCALAB250Xi, Thermo Fisher Scientific, Waltham, MA, USA), a field emission scanning electron microscope (FESEM, Zeiss Ultra Plus, Zeiss, Jena, Germany), and the contact angle test system (JC2000C, Shanghai Zhongchen Digital Technology Equipment Co., Shanghai, China).

## 3. Results

### 3.1. Membrane Characterization

**Figure 3:**
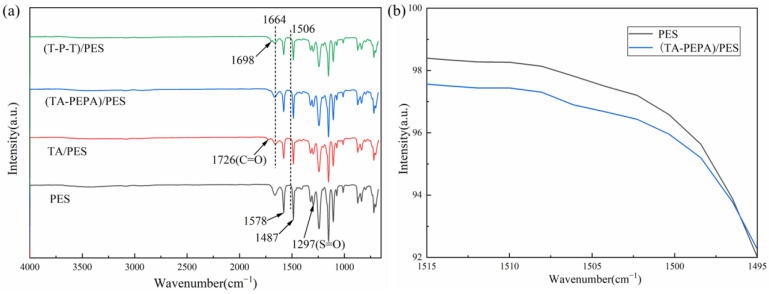
(**a**) ATR-FTIR spectra of the membrane surfaces of PES, TA/PES, (TA-PEPA)/PES, and (T-P-T)/PES membranes; (**b**) ATR-FTIR spectra of PES and (TA-PEPA)/PES membranes at 1506 cm^−1^.

Fourier transform infrared (FTIR) spectroscopy was used to characterize the surface chemical structure of the pristine and modified membranes [29]. As shown in Figure 3a, the PES membrane shows typical absorption peaks at 1578 cm^−1^ and 1487 cm^−1^, which are related to the stretching vibration of the benzene ring. The peak at 1297 cm^−1^ is the bending vibration peak of S=O in the PES membrane. After the deposition of TA, the modified membrane showed a new characteristic peak at 1726 cm^−1^, which was related to the stretching vibration of C=O, indicating that TA was successfully oxidized to the quinone group and encapsulated on the membrane surface. In addition, the absorption peak at 1664 cm^−1^ may be the overlap of the C=C resonance vibration and C=N stretching vibration in the aromatic ring. Figure 3b shows that the vibrational peak of the N-H band at 1506 cm^−1^ is present in the (TA-PEPA)/PES membrane due to the Michael addition reaction and Schiff base reaction of the TA with the single bond -NH_2_ of the PEPA [44]. In the (T-P-T)/PES membrane, a new characteristic absorption peak at 1698 cm^−1^ was observed, which was attributed to the stretching vibration of the aldehyde group.

**Figure 4:**
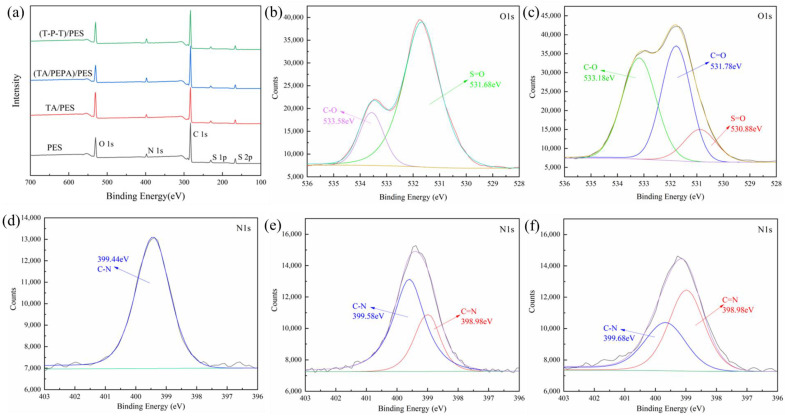
XPS spectra (**a**) of PES membrane and modified membrane; Convoluted high-resolution O1s of (**b**) PES membrane and (**c**) TA/PES membrane; Convoluted high-resolution N1s of (**d**) PES membrane and (**e**) (TA/PEPA)/PES membrane and (**f**) (T-P-T)/PES membrane.

XPS was used to characterize the content and proportion of the chemical elements on the membrane surface. The elemental composition and X-ray photoelectron spectrum of PES membranes and modified membranes are shown in Table 2 and Figure 4. After the original PES membrane is modified by TA, the content of the O element increases sharply, from the original 17.31% to 23.6%. Due to the large number of phenolic hydroxyl groups in the TA, the increase in the O element is reasonable. From Figure 4b, it can be seen that two absorption peaks of O appear in the PES membrane at 533.58 eV and 531.68 eV, which can be attributed to the C-O and S=O on the PES membrane. In Figure 4c, a new peak appears at 531.78 eV, which is attributed to C=O, while the presence of C=O indicates the self-assembly of TA on the membrane surface after oxidation. Meanwhile, the increase in the proportion of C-O peaks is attributed to the abundance of C-O groups in the TA. With the deposition of PEPA, the content of the O element decreases and the content of the N element on the membrane surface increases due to the high amount of -NH_2_ in the PEPA. These results indicate that TA-PEPA is co-deposited on the PES membrane surface. In contrast, in the (T-P-T)/PES membrane, the content of the O element is elevated, which is due to the high content of the aldehyde groups in TPAL. In addition, the N/O ratio decreases after the TA-PEPA layer is cross-linked by TPAL. Similarly, the PES membrane showed a single peak of N1s at 399.44 eV, which was related to C-N (Figure 4d). In addition, in the (TA/PEPA)/PES membrane (Figure 4e) and (T-T-P)/PES membrane (Figure 4f), the N1s could be divided into two peaks attributed to C-N and C=N, respectively. In addition, the proportion of C=N peaks increased in the (T-T-P)/PES membrane. This was consistent with the ATR-FTIR results, which further verified the reaction among TA, PEPA, and TPAL.

**Figure 5:**
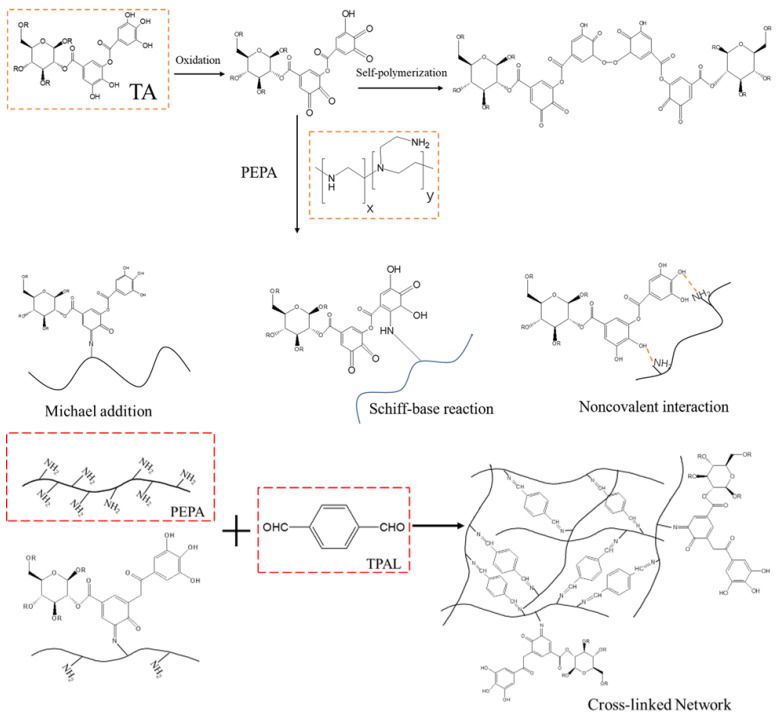
Proposed mechanism of reaction between TA, PEPA, and TPAL.

Accordingly, we propose a mechanism for the possible reaction between TA, PEPA, and TPAL based on the states between the groups that appear in the FTIR and elements in XPS [45,46]. First, TA was adsorbed on the PES ultrafiltration membrane through covalent bonds and non-covalent bonds (hydrogen bonds, π-π stacking). TA can then undergo a Michael addition reaction or Schiff base reaction with PEPA to form longer monomers that facilitate the deposition of PEPA on the membrane surface. These monomers can further undergo a Schiff base reaction with TPAL. In addition, the unreacted PEPA can also undergo a Schiff base reaction with TPAL. These two monomers can form a dense cross-linked network with TPAL that covers the PES membrane, as shown in Figure 5.

### 3.2. Morphology of the Membranes

**Figure 6:**
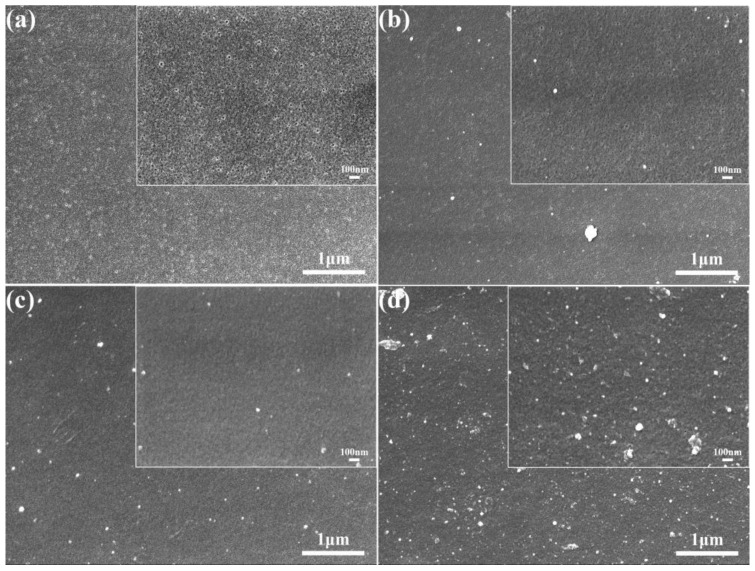
SEM images of the pristine and modified membrane surfaces. (**a**) PES pristine membrane; (**b**) TA/PES membrane; (**c**) (TA-PEPA)/PES membrane; (**d**) (T-P-T)/PES membrane.

The surface morphologies of the original PES membranes and the modified membranes are shown in Figure 6. From Figure 6a, it can be seen that the surface of the original PES membranes was relatively smooth with a large number of uniformly sized circular pores. With the self-aggregation of TA and its attachment to the membrane surface, the number of pores on the membrane surface became significantly less, and the diameter of the pores becomes smaller, which was attributed to the formation of a selective layer of TA on the membrane surface (Figure 6b). When PEPA was added, it reacted with the TA to form a dense coating and covered the membrane surface, and the pores on the membrane surface were almost invisible (Figure 6c). Figure 6d shows the electron microscope image of PEPA and TPAL after interfacial polymerization, and it can be seen that the membrane surface shows a rough texture and a large number of white dotted spheres, which cover the membrane surface relatively uniformly and form a dense selective shape layer, and the pores on the membrane surface almost completely disappear. This result also indicates the successful preparation of the modified membrane, which provided the basis for the subsequent dye separation.

### 3.3. Surface Properties of the Membranes

**Figure 7:**
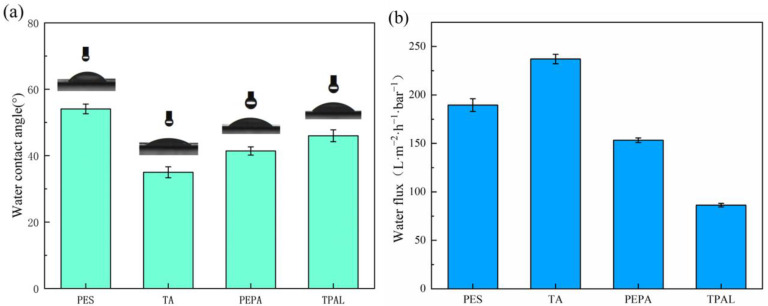
(**a**) Water contact angle of PES membrane and modified membrane; (**b**) Pure water flux of PES and modified membrane.

The static contact angles of the pristine and modified films are shown in Figure 7a. The contact angle of the pristine film was 54°, and, with the deposition of TA, the water contact angle decreased to 36°. The reason is that there are a large number of phenolic hydroxyl groups on the membrane surface, and these hydrophilic groups attract water molecules to the membrane surface through hydrogen bonding or electrostatic interaction. After modification with PEPA, the water contact angle rises slightly, which is due to the fact that the hydrophilicity of PEPA is smaller than that of TA. Therefore, when TA and PEPA are co-deposited on the surface, the water contact angle is larger than the contact angle of the membrane after depositing TA only. After the interfacial polymerization, the water contact angle increased to 44°, which was attributed to the presence of a large number of benzene rings in TPAL, which are hydrophobic groups, so the contact angle of the modified membranes increased. Figure 7b shows the pure water flux of the original membrane and the modified membrane. The water flux shows a trend of increasing first and then decreasing. This is because a small amount of TA is deposited on the PES membrane, and there are a large number of hydrophilic groups present that do not cause clogging of the membrane pores, so the water flux increases. After the addition of PEPA and TPAL, the water flux of the modified membrane gradually decreased, which was attributed to the gradual formation of a selective layer covering the membrane surface.

### 3.4. Separation Performance of the Membranes

**Figure 8:**
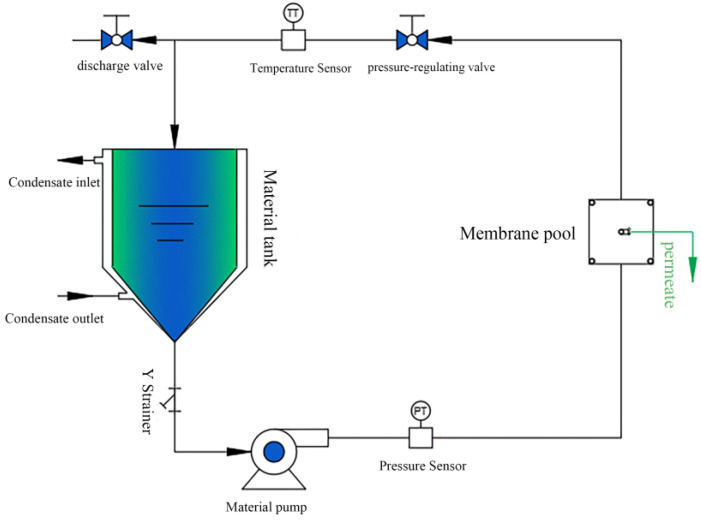
The flat film performance testing device for the separation experiment of membranes. The effective contact separation membrane area of the membrane cell was 15.9 cm^2^. The temperature was room temperature.

The flat plate membrane performance test setup is shown in Figure 8. Filtration experiments were conducted on a staggered cross-flow filtration system with an effective membrane area of 15.9 cm^2^. All the filtration experiments were performed at a pressure of 2.5 bar. The membranes were pre-pressurized in pure water for 30 min to achieve stable permeation of pure water before performing the filtration experiments. The membrane permeability was calculated by measuring the volume of the filtered solution, and the dye removal rate of the membrane was calculated by measuring the absorbance of the solution before and after filtration.

The membrane permeance was determined by the equation:

(1) $$J=\frac{V}{A\cdot t\cdot ∆p}$$

where *J* (L·m^−2^·h^−1^·bar^−1^) is the permeance, *V* (L) is the volume of the penetrating flow, *A* (m^2^) is the effective area of the membrane, *t* (h) is the filtration time, and Δ*p* (bar) is the test pressure.

The rejection rate of the dye and salt solution was determined by the equation:

(2) $$R=\left(1-\frac{C_{p}}{C_{f}}\right)\times 100\%$$

where R is the rejection of the dye molecule and *C_p_* and *C_f_* are the concentration of the permeate and the feed solution, respectively. The concentration of the dye solution was determined by a UV-Vis spectrophotometer (UV-2550, Shimadzu Corporation, Kyoto, Japan).

**Figure 9:**
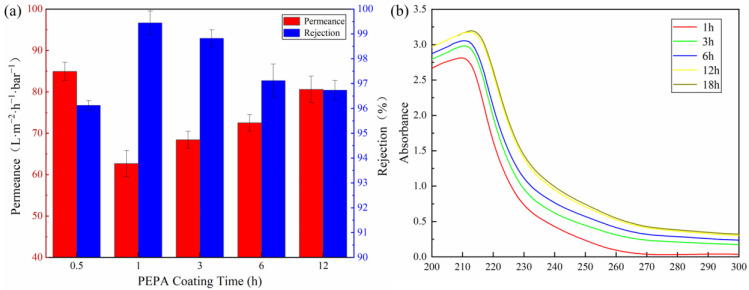
(**a**) Effect of PEPA deposition time (TPAL concentration was 0.6 g·L^−1^, interfacial reaction time was 30 min, curing time was 5 min); (**b**) UV spectra of PEPA solution, which was soaked with TA/PES membrane for different times.

Since the coating parameters have a great influence on the surface properties of membranes, the effect of the coating parameters on the membrane separation performance was investigated in detail by a cross-flow filtration system in this study. The effect of the PEPA deposition time on the separation performance of the (T-P-T)/PES membranes is shown in Figure 9a. When the fixed TPAL concentration was 0.6 g·L^−1^, the interfacial reaction time was 30 min, and the curing time was 5 min. It was found that with an increase in the PEPA deposition time, the permeance of the (T-P-T)/PES membrane first decreased and then increased, while the rejection of methyl blue was the opposite. The removal rate of methyl blue reached the maximum when the PEPA deposition time was 1 h. Generally speaking, with an increase in time, the thicker the selective layer formed by the modified substance on the membrane surface, the greater the membrane permeability resistance, the smaller the permeance, and the higher the rejection. However, the experimental results show that to be the opposite. This is because covalent bonds are formed by the addition reaction between TA and PEPA, while TA is deposited on the PES membrane in the form of hydrogen bonding and π-π conjugation. The force between the covalent bonds is much greater than that of hydrogen bonding and π-π conjugation. As a result, part of the TA on the PES film is shed and dissolved in the PEPA solution. Therefore, we analyzed the TA/PES membrane immersed in PEPA solution at different times by a UV-Vis spectrophotometer, and the results exactly illustrated this point. As can be seen in Figure 9b, there is a significant red shift in the UV spectrum with increasing immersion time, which is due to the increase in chromophores (C=O, C=N) in the solution [47]. After weighing up the advantages and disadvantages of the retention and water flux, a deposition time of 1 h was chosen as the parameter for subsequent experiments.

**Figure 10:**
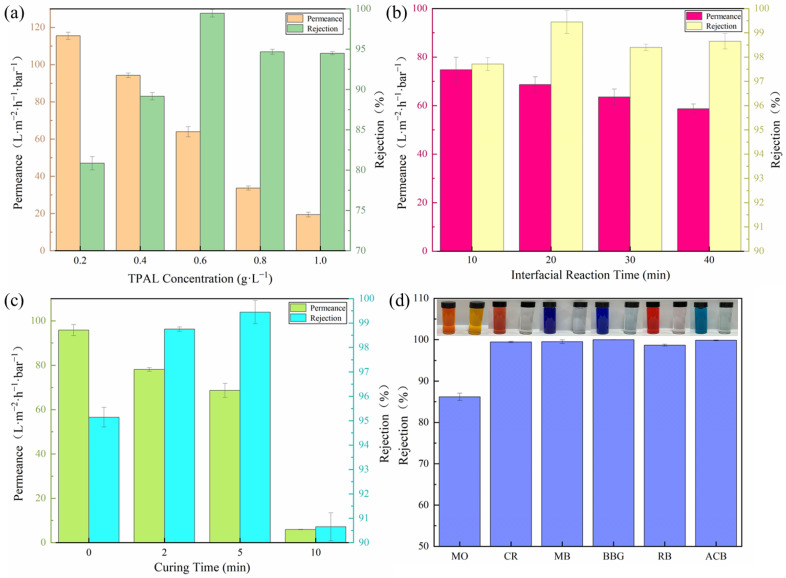
(**a**) Effect of TPAL concentration (PEPA deposition time was 1 h, interface reaction time was 30 min, curing time was 5 min); (**b**) Effect of interfacial reaction time (fixed TPAL concentration was 0.6 g·L^−1^, PEPA deposition time was 1 h, curing time was 5 min); (**c**) Effect of curing time (fixed TPAL concentration was 0.6 g·L^−1^, PEPA deposition time was 1 h, and the interfacial reaction time was 20 min); (**d**) The effect of (T-P-T)/PES membranes on various dye retention rates.

The effect of the TPAL concentration on the separation performance of the (T-P-T)/PES membranes is shown in Figure 10a. When the deposition time of the PEPA was 1 h, the interface reaction time was 30 min, and the curing time was 5 min. With an increase in the TPAL concentration, the permeance gradually decreased, and the rejection of methyl blue gradually increased. This is attributed to the fact that too low a monomer concentration would result in too thin a selective layer, while too high a monomer concentration would result in a too thick a selective layer. Therefore, a TPAL concentration of 0.6 g·L^−1^ was selected as the subsequent test condition.

The effect of the interfacial reaction time on the separation performance of the (T-P-T)/PES membranes is shown in Figure 10b. With an increase in the interface reaction time, the permeance decreased from 74.76 L·m^−2^·h^−1^·bar^−1^ to 58.68 L·m^−2^·h^−1^·bar^−1^, and the rejection of methyl blue reached a maximum of 99.45% at the interface reaction time of 20 min. The main reason for the gradual decline in the permeance is that the cross-linking degree of the two-phase monomer increases with an increase in the contact time, the formed selective layer is denser, and the membrane permeability resistance increases. When the reaction time is 20 min, the permeation and rejection are ideal because a relatively thin and loose selective separation layer is formed. Therefore, considering the comprehensive properties of the modified membrane, an interface reaction time of 20 min was selected as the subsequent test condition.

Figure 10c shows the effect of the curing time after interfacial polymerization on the separation performance of the (T-P-T)/PES membranes. When the deposition time of the immobilized PEPA was 1 h, the concentration of TPAL was 0.6 g·L^−1^, and the interfacial reaction time was 20 min. The permeance of the (T-P-T)/PES membranes decreased gradually with the prolongation of the curing time, and the methyl blue rejection showed a trend of increasing first and then decreasing. This is because a proper curing time is conducive to the formation of a defect-free modified membrane, that is, curing at 40 °C for 2–5 min is beneficial for the movement of polymer segments on the surface of the membrane. The unreacted reactive monomer will move to the interface, and a further cross-linking reaction will occur, resulting in a denser cross-linked network. In addition, when the curing time was increased from 5 min to 10 min, both the rejection and the permeation showed a sharp decrease. This is because too long a curing time causes pore size shrinkage, collapse, and even deformation of the base membrane and the selective layer. When the deformation of the two is too large, the selective layer may fall off on the surface of the base membrane, causing the performance of the (T-P-T)/PES film to drop dramatically. Therefore, after comprehensively considering the permeation and rejection of the (T-P-T)/PES membranes, a curing time of 20 min was selected as the subsequent test condition.

The separation performance of the membranes determines their application environment. According to the above exploration of experimental parameters, the optimal parameters for preparing (T-P-T)/PES membranes have been determined, that is, the deposition time of PEPA is 1 h, the concentration of TPAL is 0.6 g·L^−1^, and the interface reaction time was 20 min, and the membranes were cured at 40 °C for 5 min, and the prepared membranes had the best performance. This was also used in subsequent experiments.

In order to explore whether the best-performing membrane also has a repelling effect on different dyes, we selected dyes with different molecular weights and different charges to test the separation performance of the membranes. Figure 10d shows the separation efficiency of (T-P-T)/PES membranes for different types of dyes. The properties of the dyes can be seen in Table 1. The rejection of the (T-P-T)/PES membranes for CR, MB, BBG, RB, and ACB are all above 99%. The rejection of MO is about 86%. This may be because the molecular weight of MO is small, and MO can easily pass through the membrane’s surface. Meanwhile, the charge effect also affects the filtration performance of the membrane. Take the anionic dye CR and cationic dye ACB as an example. Due to the presence of TA in the modified membrane, it will be negatively charged in water, and according to the principle of electrostatic repulsion, the modified membrane will have high rejection of the CR dye. Meanwhile, due to the presence of PEPA in the modified membrane, the -NH_2_ on its surface will be protonated in water, forming -NH_3_^+^, which is positively charged, and thus, the modified membrane will have high rejection of the cationic ACB dye. The combination of the above two reasons leads to the high retention rate of the modified membranes for different dyes.

To highlight the outstanding properties of the membranes prepared in this work, we compared the dye rejection of the (T-P-T)/PES membrane with the results reported in other studies. As shown in Table 3, most of the reported membranes have relatively low water permeability (<50 L·m^−2^·h^−1^·bar^−1^) and dye rejection higher than 90%. With comparable dye rejection, our (T-P-T)/PES membrane exhibited excellent water permeability, which was higher than that in most of the literature. We attribute this excellent performance of the membrane to our prepared nanofiltration layer, which provides lower resistance to water transport.

### 3.5. Reusability and Long-Term Stability of the Membranes

**Figure 11:**
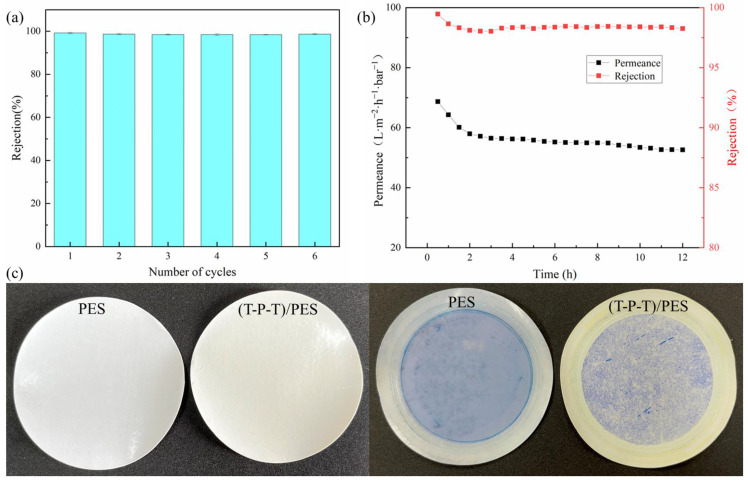
(**a**) Recyclability of (T-P-T)/PES membranes for separation of methyl blue dye; (**b**) long-term separation performance of (T-P-T)/PES membranes for methyl blue dye; (**c**) PES membrane and (T-P-T)/PES membrane before and after methyl blue dye filtration.

To further evaluate the practicality of the (T-P-T)/PES composite membranes, the reusability of the membranes was characterized by filtering the methyl blue solution. As shown in Figure 11a, with an increase in the number of cycles, the rejection of the modified membranes was almost stable and remained above 98%. This indicates that the (T-P-T)/PES composite membranes can remove surface contaminants by simple washing, and the membrane pores of the modified membranes have a certain stability.

The long-term separation performance of the (T-P-T)/PES membranes was characterized by filtration of methyl blue solution. As shown in Figure 11b, after 12 h of filtration, the rejection of methyl blue by the membranes was stable and remained above 98%. The permeation of the modified membranes decreased in the initial stage of filtration, but with an increase in the filtration time, the permeation was almost constant. These results show that the membrane has good stability during long-term filtration while still maintaining good separation performance.

In addition, as shown by the before and after photos of the methyl blue dye filtration (Figure 11c), the membrane contamination was reduced, and less dye was adsorbed on the membrane surface. The UV absorption curves of the methyl blue dye feed solution, permeate, and retentate are reported in Figure S1 of the support material. The significant increase in the concentration of the retained material indicates that the repulsion effect rather than the adsorption effect plays a dominant role in the separation of the dye molecules.

## 4. Conclusions

In this study, a novel high-throughput nanofiltration layer was prepared on a PES ultrafiltration membrane by interfacial polymerization technology for dye separation. The high throughput nanofiltration layer for dye separation was successfully prepared by the interfacial polymerization reaction of PEPA and TPAL assisted by TA. TA can be adsorbed on the PES ultrafiltration membrane in the form of covalent and non-covalent bonds (hydrogen bonding, π-π stacking) and can be used as a binder to increase the aggregation of PEPA on the membrane surface. The interfacial reaction time between PEPA and TPAL is long and easy to control. The performance of the selective layer can be adjusted by changing the coating parameters. The best performance of the modified membrane was achieved when the PEPA deposition time was 1 h, the interfacial reaction time was 20 min, and the curing time was 5 min at 40 °C. The permeate volume was 68.68 L·m^−2^·h^−1^·bar^−1^ and the rejection of methyl blue was greater than 99%. The prepared nanofiltration membranes had good separation efficiency and the rejection of various dyes (CR, MB, BBG, RB, ACB) was above 99%. Meanwhile, the modified membranes have good stability and long-term separation performance. Therefore, the modified membranes have good prospects for application in dye wastewater treatment. It is of great significance for membrane separation to improve membrane performance efficiently and precisely by designing monomers.

## Figures and Tables

**Table 1:** Properties of the organic dyes used in this study.

Dye	Structure	Molecular Weight (g·mol^−1^)	Max Absorption
Methyl orange (MO)	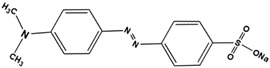	327	466
Methyl blue (MB)	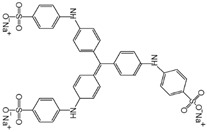	799.8	573
Congo red (CR)	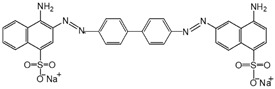	697	498
Rose red sodium salt (RB)	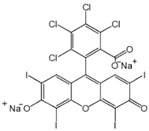	1017	508
Brilliant blue G (BBG)	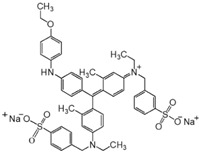	826	590
Alcian blue 8GX (ACB)	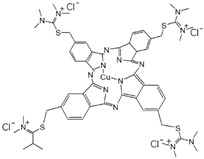	1298	620

**Table 2:** Elemental compositions of PES and modified membranes.

Membrane	Composition (Atomic %)			
	C	N	O	S
PES	73.86	4.05	17.31	4.78
TA/PES	68.82	3.67	23.60	3.90
(TA-PEPA)/PES	75.94	5.23	15.21	3.61
(T-P-T)/PES	72.51	5.55	18.09	3.81

**Table 3:** Comparison of dye filtration performance.

Membrane	Dye	Dye Rejection (%)	Flux (L·m^−2^·h^−1^·bar^−1^)	Reference
Nano-BC/Kevlar	Reactive blue 19 Methyl blue	98.2 94.7	23.2	[48]
GO/MB	Methyl orange Disperse black 9 Rhodamine B	93.32 99.85 82.56	7.67	[49]
SM/TMC	Direct red 23 Congo red	99.7 99.6	23.5	[50]
PVA-GA/Cu(OH)_2_/PESU	Congo red	98.91	37.56	[51]
Ca/GO-SA_3_	Congo red Crystal violet Methylene blue	99	38.9	[52]
TA-PEI/PES	Congo red	99.5	42.6	[49]
MIL-101(Fe)/EVAL	Chlorazol black T Congo red Eriochrome black T	99.8 99.7 98.4	43.2	[53]
TiO_2_-HMDI/PES/β-CD	Congo red Methyl blue	97.4 99.1	26	[54]
GO/HOTiO_2_/CNS@LDH	Congo red Methylene blue Crystal violet	98.42 99.84 99.41	91.15	[55]
CQD-NH_2_/PIP/TMC	Rose bengal Alcian blue	90 99	38.44	[56]
QPEI-TMC	Leucocrystal violet Rhodamine B Semixylenol orange Acid red 94	98	8.21	[57]
This work	CR, MB, BBG, RB, and ACB	99	68.68	

## Data Availability

Not applicable.

## Supplementary Materials

The following are available online at https://www.mdpi.com/article/10.3390/polym15092018/s1, Figure S1: Ultraviolet-visible absorption spectra of methyl blue dye molecules.
