# Supplementary material for: Interfacial Polymerization on Polyethersulfone Ultrafiltration Membrane to Prepare Nanofiltration Layers for Dye Separation
Source: Polymers (Basel). 2023 Apr 24;15(9):2018. doi: 10.3390/polym15092018 (PMC10181385; doi:10.3390/polym15092018)
Supplement: Supplementary file 1 [file polymers-15-02018-s001.zip › polymers-2281500-supplementary.pdf]

Support information

Interfacial Polymerization on Polyethersulfone Ultrafiltration Membrane to Prepare Nanofiltration Layers for Dye Separation

Lulu Liu [1], Weilin Wu [2], Xiaogang Jin [1,*], Xiong Luo [1], and Lili Wu [1,*]

[1] School of Materials Science and Engineering, Wuhan University of Technology, Wuhan 430070, China; 303786@whut.edu.cn (L.L.); Wuweilin1005@163.com (W.W.); 1576064780@qq.com (X.L.)

[2] School of Pharmaceutical Sciences, Hunan University of Medicine, No.492 South Jinxi Road, Huaihua Hunan 418000 P.R. China

* Correspondence: polym_jx@whut.edu.cn (X.J.); polym_wl@whut.edu.cn (L.W.)

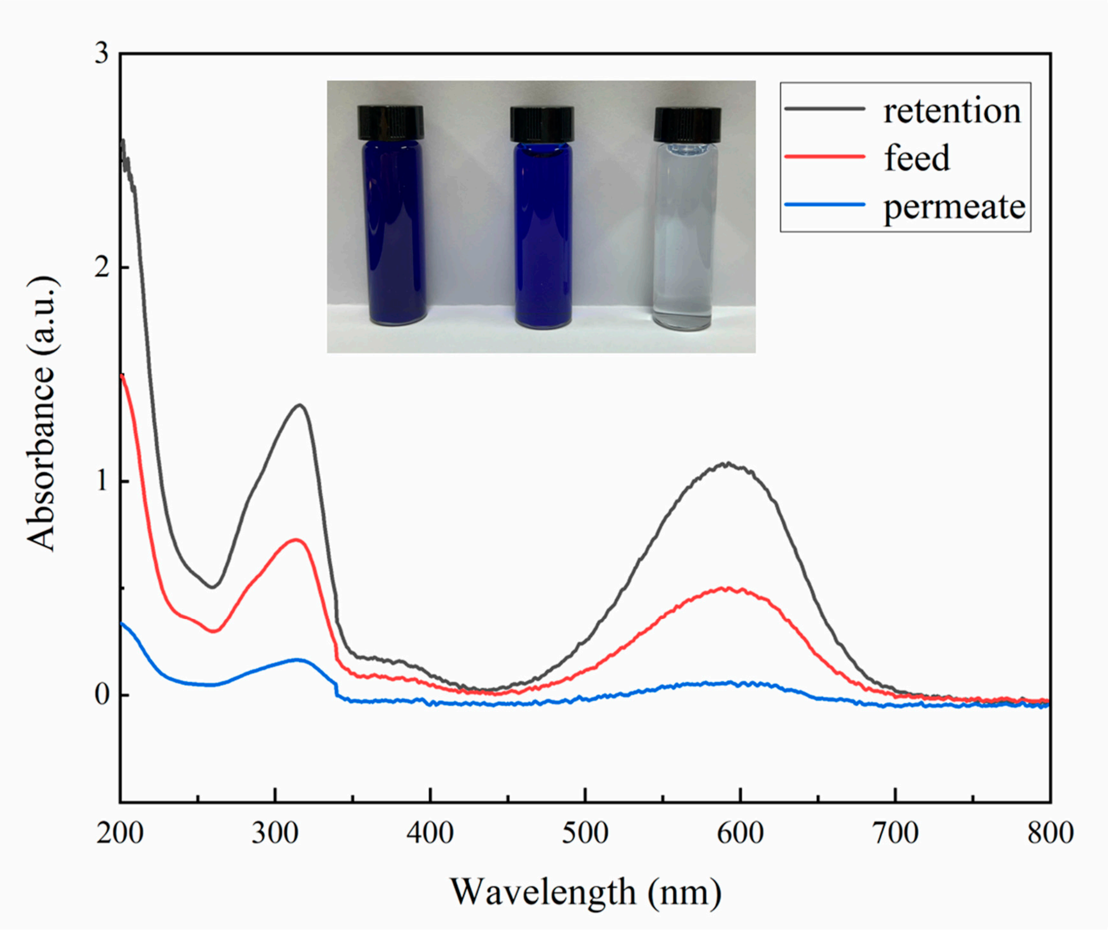

Figure S1. Ultraviolet-visible absorption spectra of methyl blue dye molecules.
